# A two-layer elasto-visco-plastic rheological model for the material parameter identification of bone tissue

**DOI:** 10.1007/s10237-020-01329-0

**Published:** 2020-05-06

**Authors:** Andreas G. Reisinger, Martin Frank, Philipp J. Thurner, Dieter H. Pahr

**Affiliations:** 1 Division Biomechanics, Department of Anatomy and Biomechanics, Karl Landsteiner University of Health Sciences, Krems an der Donau, Austria; 2 Institute of Lightweight Design and Structural Biomechanics, Vienna University of Technology, Vienna, Austria

**Keywords:** Two-layer rheological model, Trabecular bone, Single trabecula, Plasticity, Viscosity, Material parameter identification, Optimization

## Abstract

The ability to measure bone tissue material properties plays a major role in diagnosis of diseases and material modeling. Bone’s response to loading is complex and shows a viscous contribution to stiffness, yield and failure. It is also ductile and damaging and exhibits plastic hardening until failure. When performing mechanical tests on bone tissue, these constitutive effects are difficult to quantify, as only their combination is visible in resulting stress–strain data. In this study, a methodology for the identification of stiffness, damping, yield stress and hardening coefficients of bone from a single cyclic tensile test is proposed. The method is based on a two-layer elasto-visco-plastic rheological model that is capable of reproducing the specimens’ pre- and postyield response. The model’s structure enables for capturing the viscously induced increase in stiffness, yield, and ultimate stress and for a direct computation of the loss tangent. Material parameters are obtained in an inverse approach by optimizing the model response to fit the experimental data. The proposed approach is demonstrated by identifying material properties of individual bone trabeculae that were tested under wet conditions. The mechanical tests were conducted according to an already published methodology for tensile experiments on single trabeculae. As a result, long-term and instantaneous Young’s moduli were obtained, which were on average 3.64 GPa and 5.61 GPa, respectively. The found yield stress of 16.89 MPa was lower than previous studies suggest, while the loss tangent of 0.04 is in good agreement. In general, the two-layer model was able to reproduce the cyclic mechanical test data of single trabeculae with an root-mean-square error of 2.91 ± 1.77 MPa. The results show that inverse rheological modeling can be of great advantage when multiple constitutive contributions shall be quantified based on a single mechanical measurement.

## Introduction

Mechanical testing is the gold standard for obtaining bone tissue material properties on the macroscopic and mesoscopic length scale in experimental biomechanics. Material properties like stiffness and strength condense the material response to applied stress or strain into descriptive values. These values allow for easy comparison of sample groups (young vs. old or healthy vs. pathological, etc.) and are used as an input for constitutive material models.

Bone is a living tissue that is subjected to continuous adaptations. Its mechanical behavior is subjected to changes over time due to a remodeling process (Lanyon et al. 1982; Giorgio et al. 2016, 2017). Mechanical testing requires usually to extract bone samples from the living environment or utilizes donor tissue where remodeling and metabolic processes have stopped. As such, mechanical testing generally provides only a still image of the continuous tissue alterations that take place in the living organism.

As known for many decades, the material response of compact bone to a mechanical load includes multiple constitutive effects. Aside its well-investigated linear elastic stiffness, bone tissue also shows a viscous contribution (McElhaney 1966). In the post-yield regime, bone is hardening (Reilly et al. 1974), while stiffness is degrading due to microstructural damage (Zioupos and Currey 1994; Garcia 2006; Leng et al. 2009).

For separating these effects in mechanical tests, appropriate loading protocols must be used. To quantify viscosity, tests at different strain rates, sinusoidal, relaxation, or creep tests have to be performed (Lakes et al. 1979; Sasaki et al. 1993; Yamashita et al. 2001; Abdel-Wahab et al. 2011). The post-yield behavior is assessed with monotonic or cyclic loading till failure (Reilly and Burstein 1975; Carter et al. 1981; Pattin et al. 1996). It is very demanding to identify elastic, inelastic and viscous contributions on a basis of a single specimen, as fundamentally different tests have to be done in succession. Care must be taken to keep the specimen perfectly intact from test to test. This means, that mechanical damage must be avoided and also biological degradation of the samples has to be inhibited. Moreover, testing different mechanical properties on multiple sets of samples requires statistical methods to compensate for the high inter-sample variation that is inherent for biological samples.

Mechanical properties are typically obtained by extracting them directly from stress and strain data. Hereby, different practical approaches exist. One method for determining the yield stress uses the last point of the linear region, with the linear region being found by the $R^2$ method (Synek et al. 2015; Frank et al. 2018). Other methods use the 0.2% strain limit (Keaveny et al. 1994a), or a line intersection method (Reilly and Burstein 1975). Those methods very likely produce different results, which is problematic when determining an intrinsic property of the material. Another problem that is often accepted for the sake of simplicity concerns the viscoelasticity of bone: As bone is viscoelastic, the young’s modulus and yield stress extracted from stress–strain curves are depending on the applied strain rate. In general, those quantities represent apparent properties and not intrinsic properties of the material.

Especially, on the submillimeter length scale of single bone structural units like trabeculae, the determination of material properties is still a challenge (Lucchinetti et al. 2000; Carretta et al. 2013a; Hernandez et al. 2005; Bini et al. 2002; Szabó et al. 2011a, b; Jungmann et al. 2011). When keeping bone samples out of the living environment, degradation and dehydration processes start. To determine bone’s material properties close to an in vivo state, bone should be tested fresh and be kept wet. Recent advantages in testing procedures allow for testing individual trabeculae under wet (submerged) conditions in tension (Frank et al. 2017, 2018). These delicate tests are laborious, have a considerable reject rate and are prone to noisy data so every successfully tested sample is precious. Viscous and post-yield material properties of single trabeculae are yet to be determined. As the individual strut represents the structural unit of trabecular bone, knowledge of its material properties, that is not only stiffness but also viscous and post-yield properties is of great importance in the diagnosis of bone related diseases like osteoporosis and fracture risk estimation. Classically such material properties have not been directly measured (except in nanoindentation tests, e.g., Polly et al. 2012; Donnelly et al. 2006) but inferred from combined mechanical testing and finite element simulations (Mullins et al. 2009; Carnelli et al. 2010).

An alternative approach for determining a set of material parameters from stress–strain data of a bone specimen is the method of parameter identification by optimization (also referred to as ’inverse method’). Hereby, the parameters of a suitable material model are optimized so that the model response coincides with the mechanical testing data (Muller and Hartmann 1989; Ichikawa and Ohkami 1992; Gelin and Ghouati 1995). The found set of parameters are then supposed to represent the material parameters of the tested sample. The underlying material model needs to contain formulations for the constitutive effects that should be identified. Also the test protocol must allow to assess the different constitutive effects included in the material model. At the same time, the number of material parameters must be kept low to ease the optimization process and to avoid overfitting and ambiguous solutions.

In the case of uniaxial stress, simplified one dimensional rheological model representations are practical, where elements of certain constitutive effects are combined in series or parallel (Sperry 1964). This way of describing material behavior is commonly used in well known material models for bone on the tissue level, that incooporate combinations of viscous and plastic and damage contributions (Garcia 2006; Garcia et al. 2010; Schwiedrzik et al. 2014; Schwiedrzik 2014; Natali et al. 2008; Fondrk et al. 1999).

In this study, we propose a two-layer elasto-visco-plastic rheological model for reproducing the uniaxial behavior of small length-scale bone samples. The two-layer model topology is based on the work of Kichenin (1992) and extended by an exponential hardening law going back to Voce (1948). To the authors knowledge, the model configuration presented in this work, has not yet been applied to bone tissue. The two-layer model is used for the identification of stiffness, damping, yield stress and hardening coefficients of individual human bone trabeculae, based on cyclic tensile test data obtained in wet conditions. Hereby the models’ material parameters are optimized with a multi-start method so that the model response follows the experimentally obtained stress–strain data of an individual bone trabecula.

## Methods

### The two-layer elasto-visco-plastic rheological model

Besides being linear elastic, bone’s response to a mechanical load includes different energy dissipation mechanisms. Beyond the yield point, bone is exhibiting plastic flow (Reilly et al. 1974) and damage (Zioupos and Currey 1994). In addition, its mechanical behavior is depending on strain rate and thus contains a viscous contribution (McElhaney 1966).

The two-layer rheological model described in the following, aims to capture the plastic and viscous behavior of bone tissue in order to model the mechanical response of single bone trabeculae under cyclic tensile load. (The constitutive effect of damage is neglected in this work, which will be justified in hindsight in Sec. 4.)

**Fig. 1:**
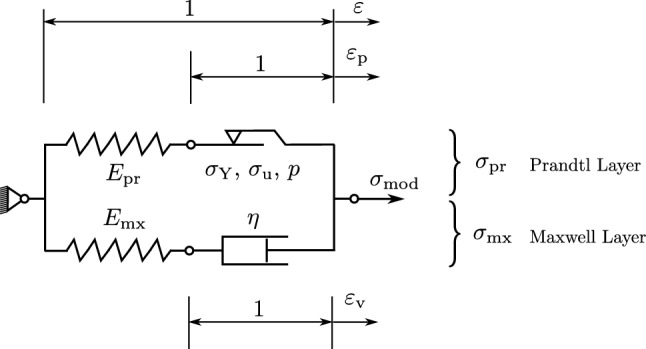
Two-layer rheological model, consisting of a Prandtl layer and a Maxwell layer in parallel with the associated layer stresses $\sigma _{\mathrm{pr}}$ and $\sigma _{\mathrm{mx}}$, respectively. The strain in the plastic slider is denoted as $\varepsilon _{\mathrm{p}}$ and the strain in the damper as $\varepsilon _{\mathrm{v}}$. $E_{\mathrm{pr}}$, $E_{\mathrm{mx}}$ are the elastic moduli of the springs, $\sigma _{\mathrm{Y}}$, $\sigma _{\mathrm{u}}$ are yield- and ultimate stress, *p* is the exponential hardening exponent and $\eta$ the damping parameter. The model’s global state is described by the global stress $\sigma _{\mathrm{mod}}$ and global strain $\varepsilon$

The two-layer rheological model consists of a Prandtl model and a Maxwell model arranged as two parallel layers, Fig. 1. The total model stress $\sigma _{\mathrm{mod}}$ is the sum of the stress $\sigma _{\mathrm{pr}}$ in the Prandtl layer and the stress $\sigma _{\mathrm{mx}}$ in the Maxwell layer.

1 $$\begin{aligned} \sigma _{\mathrm{mod}} = \sigma _{\mathrm{pr}} + \sigma _{\mathrm{mx}} \end{aligned}$$

and in terms of stress rates,

2 $$\begin{aligned} {\dot{\sigma }}_{\mathrm{mod}} = {\dot{\sigma }}_{\mathrm{pr}} + {\dot{\sigma }}_{\mathrm{mx}} \end{aligned}$$

*Prandtl Layer* The Prandtl layer itself is built from an elastic spring with elastic modulus $E_{\mathrm{pr}}$ in series with a plastic slider (Sperry 1964; Grzesikiewicz and Zbiciak 2012). The total strain $\varepsilon$ of the two-layer model splits here into an elastic part and in a plastic part $\varepsilon _{\mathrm{p}}$ resulting in the elastic relationship

3 $$\begin{aligned} \sigma _{\mathrm{pr}} = E_{\mathrm{pr}} (\varepsilon -\varepsilon _{\mathrm{p}}) \end{aligned}$$

**Fig. 2:**
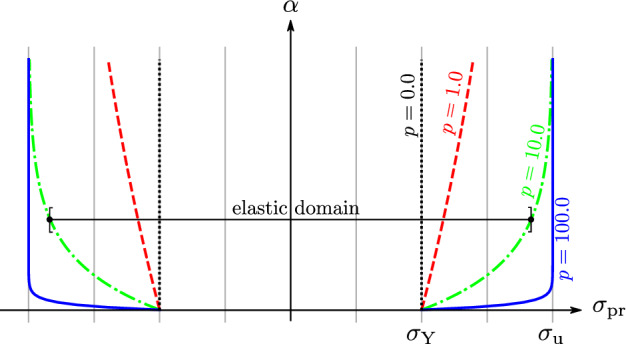
Evolution of the elastic domain of the Prandtl layer in stress space, depending on the equivalent plastic strain $\alpha$. Drawn for different values of the exponent *p*

For the plastic slider, a yield condition *f* is defined whose yield limit is expanding exponentially in the amount of equivalent plastic strain $\alpha$, based on Voce (1948).

4 $$\begin{aligned} f(\sigma _{\mathrm{pr}},\alpha ) = |\sigma _{\mathrm{pr}}| - [\sigma _{\mathrm{Y}}+(\sigma _{\mathrm{u}}-\sigma _{\mathrm{Y}})(1-\exp (-\alpha p))] \end{aligned}$$

with $\exp (x)=e^x$. In this approach, the stress is converging against an ultimate stress $\sigma _{\mathrm{u}}$ with increasing $\alpha$. The onset of plastic deformation starts at the yield stress $\sigma _{\mathrm{Y}}$ of the material. The exponent *p* is shaping the stress evolution between $\sigma _{\mathrm{Y}}$ and $\sigma _{\mathrm{u}}$, Fig. 2.

The basic idea is that plastic flow in the sense of $|\dot{\varepsilon }_{\mathrm{p}}|>0$ occurs only if a stress state $\sigma _{\mathrm{pr}}$ reaches the current yield limit where $f=0$. Stress states $\sigma _{\mathrm{pr}}$ for which $f<0$ are elastic and no change in $\varepsilon _{\mathrm{p}}$ takes place and thus $\dot{\varepsilon }_{\mathrm{p}}=0$.

The evolutionary equation for $\alpha$ is simply

5 $$\begin{aligned} \dot{\alpha } = |\dot{\varepsilon }_{\mathrm{p}} | \end{aligned}$$

The flow rule is defined as

6 $$\begin{aligned} \dot{\varepsilon }_{\mathrm{p}} = \gamma \ \mathrm{sign}(\sigma _{\mathrm{pr}}) \end{aligned}$$

with the function $\gamma$ being the slip rate.

Stress $\sigma _{\mathrm{pr}}$ and $\gamma$ are restricted by the *Kuhn–Tucker complementary conditions* (Simo 1998): First, $\sigma _{\mathrm{pr}}$ needs to be admissible and reside within or on (but not outside) the yield surface. In addition, plastic flow must go into the direction of the applied stress, which implies $\gamma$ to be nonnegative. Consequently,

7 $$\begin{aligned} f(\sigma _{\mathrm{pr}},\alpha ) \le 0 \quad \mathrm{and} \quad \gamma \ge 0 \end{aligned}$$

Second, it is required that plastic flow occurs only for stress states residing on the yield surface and that stress states which reside within the yield surface do not lead to plastic flow.

8 $$\begin{aligned} \gamma f(\sigma _{\mathrm{pr}},\alpha ) = 0 \end{aligned}$$

For $\dot{\varepsilon }_{\mathrm{p}}$ being nonzero, the stress point must *persist* on the yield surface so that $\dot{f}(\sigma _{\mathrm{pr}},\alpha ) = 0$ for $\gamma > 0$ (Simo 1998). This adds the *persistency* (or *consistency*) condition

9 $$\begin{aligned} \gamma \dot{f}(\sigma _{\mathrm{pr}},\alpha ) = 0 \quad \mathrm{(if} \quad f(\sigma _{\mathrm{pr}},\alpha ) = 0) \end{aligned}$$

From the consistency condition (Eq. 9), it follows that $\gamma$ can be nonzero only if

10 $$\begin{aligned} \dot{f} = \frac{\partial f}{\partial \sigma _{\mathrm{pr}}} {\dot{\sigma }}_{\mathrm{pr}} + \frac{\partial f}{\partial \alpha } {\dot{\alpha }} = 0 \end{aligned}$$

From Eq. 10 and substituting Eqs. 3, 4, 5, 6 it is possible to solve for the slip rate $\gamma$ in case of plastic flow $(f(\sigma _{\mathrm{pr}},\alpha ) = 0)$. Together with the Kuhn–Tucker condition (Eq. 8), one obtains the expressions for the slip rate

11 $$\begin{aligned} \gamma = {\left\{ \begin{array}{ll} \mathrm{sign}(\sigma _{\mathrm{pr}}) \frac{E_{\mathrm{pr}} \dot{\varepsilon }}{E_{\mathrm{pr}}+(\sigma _{\mathrm{u}}-\sigma _{\mathrm{Y}}) p \exp (-p \alpha )},&\quad \text {if } f(\sigma _{\mathrm{pr}},\alpha ) = 0\\ 0 ,&\quad \text {if } f(\sigma _{\mathrm{pr}},\alpha ) < 0 \end{array}\right. } \end{aligned}$$

*Maxwell layer* The Maxwell layer is built from an elastic spring with elastic modulus $E_{\mathrm{mx}}$ in series with a viscous damper with coefficient of viscosity $\eta$. Its well-known governing equation takes the form (Marques and Creus 2012)

12 $$\begin{aligned} {\dot{\sigma }}_{\mathrm{mx}} + \frac{E_{\mathrm{mx}}}{\eta } \sigma _{\mathrm{mx}} = E_{\mathrm{mx}} {\dot{\varepsilon }} \end{aligned}$$

The damper strain $\varepsilon _{\mathrm{v}}$ is obtained by

13 $$\begin{aligned} \varepsilon _{\mathrm{v}} = \varepsilon - \frac{\sigma _{\mathrm{mx}}}{E_{\mathrm{mx}}} \end{aligned}$$

*Time integration* For a *strain-driven process*, ${\varepsilon }$ (and thus $\dot{\varepsilon }$) is a known time signal over time *t*. Then the problem to be solved consists of determining the stress response in the Prandtl layer $\sigma _{\mathrm{pr}}$ and the Maxwell layer $\sigma _{\mathrm{mx}}$ due to ${\varepsilon }$ and adding them up to $\sigma _{\mathrm{mod}}$ according to Eq. 1. Due to the topology of the model, $\sigma _{\mathrm{pr}}$ and $\sigma _{\mathrm{mx}}$ are decoupled and can be determined independently. Time integration strategies for the Prandtl layer are described in the “Appendix”. The Maxwell Layer can be solved in time domain by standard ODE solvers or by integrating the hereditary integral (Gutierrez-Lemini 2014).

*DMA properties* For comparison of the pure viscoelastic properties of the two-layer model with dynamic mechanical analysis (DMA) data from other studies, its storage modulus $E'$, loss modulus $E''$ and loss tangent $\tan (\delta )$ are derived.

For a harmonic excitation, the two-layer elasto-visco-plastic model from Fig. 1 behaves like a Zener model if operated in the elastic range. The governing equation of the viscoelastic Zener model is Marques and Creus (2012)

14 $$\begin{aligned} \sigma + \frac{\eta }{E_{\mathrm{mx}}} {\dot{\sigma }} = E_{\mathrm{pr}}\ \varepsilon + \frac{\eta (E_{\mathrm{pr}}+E_{\mathrm{mx}})}{E_{\mathrm{mx}}}{\dot{\varepsilon }} \end{aligned}$$

Applying the Laplace transform ${\mathcal{L}}()$ transfers Eq. 14 from time domain into frequency domain, which gives

15 $$\begin{aligned} {\overline{\sigma }} + a s {\overline{\sigma }} = b \varepsilon + c s {\overline{\varepsilon }} \end{aligned}$$

with the constants

16 $$\begin{aligned} \begin{aligned} a&= \frac{\eta }{E_{\mathrm{mx}}} \\ b&= E_{\mathrm{pr}} \\ c&= \frac{\eta (E_{\mathrm{pr}}+E_{\mathrm{mx}})}{E_{\mathrm{mx}}} \end{aligned} \end{aligned}$$

and *s* being the complex frequency parameter. ${\overline{\sigma }}$ and ${\overline{\varepsilon }}$ are the Laplace-transformed functions of stress and strain, respectively. For a harmonic strain excitation, the transfer function is obtained by

17 $$\begin{aligned} H(s)=\frac{{\rm output}}{{\rm input}}=\frac{{\overline{\sigma }}}{{\overline{\varepsilon }}}=\frac{b+c\ s}{1+ a\ s} \end{aligned}$$

The frequency response or *gain* of the system is obtained by evaluating *H*(*s*) at $i \omega$, where $\omega$ is the angular frequency and $i=\sqrt{-1}$. In the context of DMA, the frequency response is equivalent to the *complex modulus* $E^*$

18 $$\begin{aligned} \begin{aligned} H(i\omega )=E^*(\omega ) =E'(\omega )&+ i E''(\omega ) = \\ = \frac{a c \omega ^2 + b}{a^2 \omega ^2 + 1}&+ i \frac{c \omega - a b \omega }{a^2 \omega ^2 + 1} \end{aligned} \end{aligned}$$

From Eq. 18, the storage modulus $E'$ and loss modulus $E''$ can be easily extracted for a given angular frequency $\omega$. The *loss tangent* (or *loss factor*) is the tangent of the phase shift $\delta$ between strain excitation and stress response and given by

19 $$\begin{aligned} \tan (\delta ) = \frac{E''}{E'} = \frac{c \omega - a b \omega }{a c \omega ^2 + b} \end{aligned}$$

*Long-term and instantaneous Young’s Modulus* When loading the two-layer model quasi-statically the Maxwell layer has no stress contribution and stays fully relaxed ($\sigma _{\mathrm{mx}}=0$). Similarly, holding a certain deformation state until the viscous stress contribution is decayed also results in $\sigma _{\mathrm{mx}}=0$. In these two cases, the model stiffness is solely driven by the elastic spring in the Prandtl layer. $E_{\mathrm{pr}}$ can be therefore referred to as the *quasi-static* or *long term* Young’s modulus.

In contrast, when applying a step load on the model in the form of a Heaviside step function, the apparent model stiffness is the sum $E_{\mathrm{pr}}+E_{\mathrm{mx}}$, which is therefore referred to as the *instantaneous* Young’s modulus.

So $E_{\mathrm{pr}}$ and $E_{\mathrm{pr}}+E_{\mathrm{mx}}$ make up the lower and the upper bound in between any apparent Young’s modulus obtained at a finite strain rate must reside.

### Tensile testing of individual trabeculae

To validate the two-layer elasto-visco-plastic rheological model, microtensile tests on individual bone trabeculae were conducted. The reason for testing bone microstructural units instead of macroscopic bone samples lies in the reduced contribution of damage in the deformation mechanism (Schwiedrzik et al. 2014) and largely also in the exclusion of structural effects present in mechanical tests conducted on larger trabecular bone samples.

*Sample preparation* The usage of human tissue in this study was approved by the Southampton and SouthWest Hampshire Research Ethics Committee, ethic votes LREC 194/99/1, 210/01, 12/SC/0325. 28 individual trabeculae were dissected from the central femoral head of a 61 year old female donor. First, the central femoral head was cut into 3 slices, in the frontal plane, of 2 mm thickness with a bandsaw (300 CP – Diamond Bandsaw, Exakt, Germany). Bone marrow was removed from these slices with a dental water jet (OralB, Germany). Sequentially, a previously established dissection and processing protocol for preparation of the individual trabeculae was used (Frank et al. 2018).

**Fig. 3:**
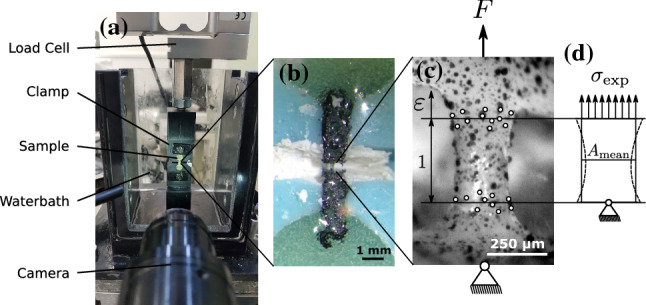
**a** Tensile test setup. **b** Embedded tensile sample. The bone trabecula resides between the two stained (black) epoxy strips. **c** Trabecula, with applied speckle pattern and tracking points at the top and bottom during tensile testing. Horizontal lines indicate the mean vertical position of the points and are used for strain calculation. **d** The representative cross section $A_{\mathrm{mean}}$ of each sample, determined from $\mu$CT scans, is used for stress calculation

This protocol aims to process several trabeculae in parallel and to obtain wet samples for testing. In brief, trabeculae were cut under a microscope (SZX10, Olympus Corporation, Japan) with a hand held miller (Dremel 400, Dremel Europe, the Netherlands). Only samples that had an aspect ratio of at least 3 were selected. After cutting, the geometry of every trabeculae was obtained with a calibrated $\mu$CT100 (Scanco Medical AG, Switzerland) at 70 kVp, 114 $\mu$A, integration time 200 ms, average data 4, 1500 projections, nominal resolution of 3.3 μm and aluminum filter 0.5 mm. Then, the ends of the trabeculae (and the adjacent bone) were embedded in epoxy glue (UHU Endfest 300, UHU, Germany) for at least 16 h in custom-made silicone chambers, in order to mount the samples properly into the tensile test device, Fig. 3a, b). After that, a speckle pattern was applied with a water-soluble spray paint (RAL9005, Dupli-Color, Motip dupli, Germany) to enable optical strain recording. Then, samples were rehydrated in Hank’s balanced saline solution, HBSS (pH = 7.4) for at least 2h at room temperature before testing. Figure 3c shows a representative sample with the applied speckle pattern, recorded with the video camera during tensile testing.

*Mechanical testing* Individual samples were mounted to a servo-electric load frame (SELmini-001, Thelkin AG, Switzerland), equipped with a custom made tensile test set-up (as presented in Frank et al. 2018). The set-up (see Fig. 3a) was modified to allow a better sample alignment. In the current configuration, the sample can be fixated with two clamps to prevent movement out of the frontal plane. The whole setup is placed in a water bath, which is filled with HBSS to mimic a physiologic environment. Strain was recorded with a camera (UI-3250CP-M-GL, IDS GmbH, Germany), at 10 Hz which was equipped with a KITO-D zoom objective (mounted on a KITO-ADP-0.5 adapter, Kitotec GmbH, Germany). Strain determination is performed with the digital image correlation (DIC) package *trackpy* (Allan et al. 2016), which detects several points at the top and at the bottom of the trabecula. These points are tracked over time and the length between the dots at the top and the bottom is determined. The change in length related to the original length yields the engineering strain $\varepsilon$ of the sample, Fig. 3c. A 10-N load cell (HBM-S2M, Germany, relative error 0.02% of full scale output) was used to measure the experimental force. The obtained mean particle positions from strain tracking at the top and the bottom of the trabeculae were used as a reference for cropping the representative volume, Fig. 3c. A representative cross-sectional area $A_{\mathrm{mean}}$ was then determined by dividing the obtained volume of each trabeculae (from the $\mu$CT, as presented in Frank et al. (2017)) by its length. With this, engineering stress was calculated by $\sigma _{\mathrm{exp}}=F/A_{\mathrm{mean}}$, where *F* is the force signal.

**Fig. 4:**
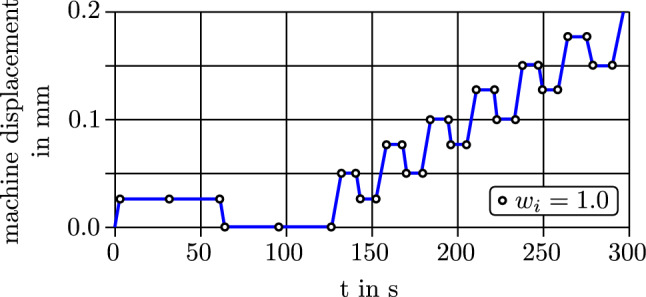
Loading profile of the trabecular sample with controlled machine displacement over time. The white circles indicate points at which the RMSE objective function is weighted with weighting factor $w_i=1.0$. $w_i=0.0$ otherwise. The apparent Young’s modulus $E_{\mathrm{app}}$ is extracted from the stress–strain data of the first loading ramp

*Loading profile* A preload of $\sim$ 0.05 N was applied and held for 30 s to align parts and close gaps within the clamps. The main loading profile was displacement-controlled and attempted to accentuate the viscous and plastic response of the sample, Fig. 4. It consisted of two parts, which both were performed at a displacement rate of 0.01 mm/s. In the first part, it was attempted to make the viscous force relaxation visible. Here, the sample was loaded up to 0.025 mm (machine displacement) and held at this position for 60 s. Then, the sample was unloaded to position 0 mm and held for 60 s. In the second part, the sample was always elongated by 0.05 mm, compared to the previous step and held for 10 s. Then, it was unloaded by 0.025 mm and held for 10 s. This procedure of loading, holding, unloading, holding continued until the sample was fractured. The obtained measurement data was resampled to 1Hz, to reduce the computational expense for solving the model.

The samples fractured at different points in time. To unify the data, the time series were cut off at the end of the fourth loading cycle. Samples that fractured before that, were excluded from the study. As the time series is used as an input for the two-layer model, the preload had to be excluded from the data by shifting load and displacement to be 0.0 at $t=0$. That was necessary to avoid an step load as model input, that would lead to an unrealistic initial viscous stress response. After optimization, $\sigma _{\mathrm{Y}}$ and $\sigma _{\mathrm{u}}$ were then corrected for the prestress originating from the applied preload.

### Material parameter identification

A set of material parameters ${\underline{q}}=[E_{\mathrm{pr}},\sigma _{\mathrm{Y}},\sigma _{\mathrm{u}},p,E_{\mathrm{mx}},\eta ]$ shall be found, for which the stress response $\sigma _{\mathrm{mod}}$ of the two-layer model fits best to the measured stress response $\sigma _{\mathrm{exp}}$ of a trabecular sample.

As $\sigma _{\mathrm{mod}}$ is calculated for the time discretization of $\sigma _{\mathrm{exp}}$, both time series are synchronized. The goodness of their fit can be expressed in terms of the weighted *root-mean-square error* ($\hbox {RMSE}_{\mathrm{w}}$) evaluated at the time points $t_i$ of the time series (with $i=1...n$ and *n* being the total number of time points).

20 $$\begin{aligned} \mathrm{RMSE}_{\mathrm{w}}({\underline{q}})=\sqrt{\frac{1}{n} \sum _{i=1}^n w_i (\sigma _{\mathrm{mod}}({\underline{q}},\varepsilon ,t_i)-\sigma _{\mathrm{exp}}(t_i))^2} \end{aligned}$$

Here, $\sigma _{\mathrm{mod}}$ denotes the two-layer model stress response, evaluated for a certain set of material parameters ${\underline{q}}$ and the strain signal $\varepsilon$ from the tensile test of a sample. The weighting factor $w_i$ enables to emphasize certain time points in the optimization process. It was found in pretests, that the trivial approach with $w_i=1.0$ at all points in time leads to a higher rate of odd or indistinct optimization results. The optimization process is more robust, when $w_i=1.0$ at the points highlighted in Fig. 4 and $w_i=0.0$ otherwise.

When setting $w_i=1.0$ for all $t_i$, the weighted RMSE simplifies to the standard RMSE (Chai and Draxler 2014; Crawley 2007), that can be interpreted as the bandwidth around the $\sigma _{\mathrm{exp}}$ signal in which 68% of the $\sigma _{\mathrm{mod}}$ signal resides.

21 $$\begin{aligned} \mathrm{RMSE}({\underline{q}})=\mathrm{RMSE}_{\mathrm{w}}({\underline{q}}) \quad | \quad w_i=1.0\ \forall \ i \end{aligned}$$

Equation 20 is taken as the objective function for the optimization task, which consists of choosing ${\underline{q}}$ so that

22 $$\begin{aligned} \mathrm{RMSE}_{\mathrm{w}}({\underline{q}})\rightarrow \mathrm{min} \quad \mathrm{with} \quad \{{\underline{q}} \in {\mathbb{R}}\ |\ {\underline{q}} > 0\} \end{aligned}$$

**Fig. 5:**
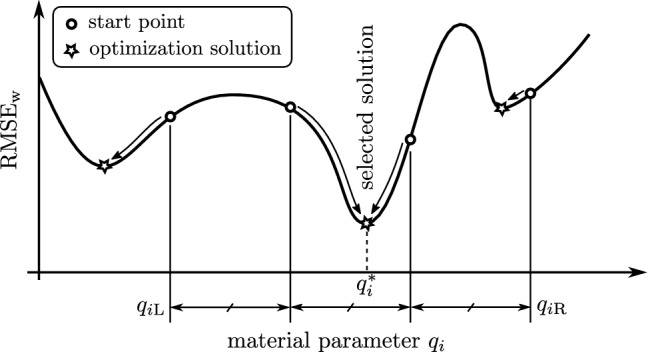
Multi-start method, exemplary shown for a single material parameter $q_i$. The optimization is started at 4 equally spaced points for which three separate local minimums in the objective function $\mathrm{RMSE}_{\mathrm{w}}$ are found. Small arrows indicate the optimization process of the downhill simplex algorithm. The $q_i$ value at the lowest minimum is selected as the ’global’ solution $q_i^*$

The optimization task from Eq. 22 is addressed with a downhill simplex algorithm (Nelder and Mead 1965). This method relies on the selection of a suitable start parameter set ${\underline{q}}$, at which the optimization is initialized. As the shape of the objective function Eq. 20 is unknown, the selection of the initial ${\underline{q}}$ is difficult and potentially crucial at the same time. It was found in pretests, that the objective function appears to have multiple local minima, which makes the solution returned by the algorithm highly dependent on the choice of this initial parameter set. To mitigate that problem, the optimization task was performed using a *multi-start method*. In particular, each material parameter $q_i$ in ${\underline{q}}$ was assigned a meaningful range $q_{i\mathrm{L}} \le q_i \le q_{i\mathrm{R}}$, based on results from Frank et al. (2018). That range was spanning one or two orders of magnitude and expected to contain (or to be close by) the global minimum of that parameter, Table 1. By subdividing each range by four points, $4^6=4096$ points in parameter space of ${\underline{q}}$ were created and used as starting points for optimization. As a result, 4096 optimization solutions were obtained, from which ${\underline{q}}^*$, the solution with the minimum $\mathrm{RMSE}_{\mathrm{w}}$ value was selected, and considered as a quasi ’global’ solution, Fig. 5. The standard RMSE value according to Eq. 21 was then calculated at ${\underline{q}}^*$ to ease result interpretation.

**Table 1:** Table containing the material parameter ranges used for the multi-start optimization method

$q_i$	$q_{i\mathrm{L}}$	$q_{i\mathrm{R}}$	Unit
$E_{\mathrm{pr}}$	500	5000	MPa
$\sigma _{\mathrm{Y}}$	10	100	MPa
$\sigma _{\mathrm{u}}$	$\sigma _{\mathrm{Y}}$+10	$\sigma _{\mathrm{Y}}$+100	MPa
*p*	10	1000	1
$E_{\mathrm{mx}}$	500	5000	MPa
$\eta$	2000	20000	MPa s

### Apparent Young’s Modulus

For the purpose of comparison with the modeling approach, the Young’s modulus of each sample is also evaluated in a classical way. Therefore linear regression is performed on the first loading cycle of the experimental stress–strain data. Starting from a few data points, the data window is enlarged while evaluating the coefficient of determination $R^2$ (Frank et al. 2018). The specific data window for which the $R^2$ reaches a maximum is taken as the linear region of the stress–strain data. The apparent Young’s modulus $E_{\mathrm{app}}$ is calculated as the slope of the linear regression in this linear region. As being an apparent property, it incorporates viscous and/or other effects.

## Results

**Fig. 6:**
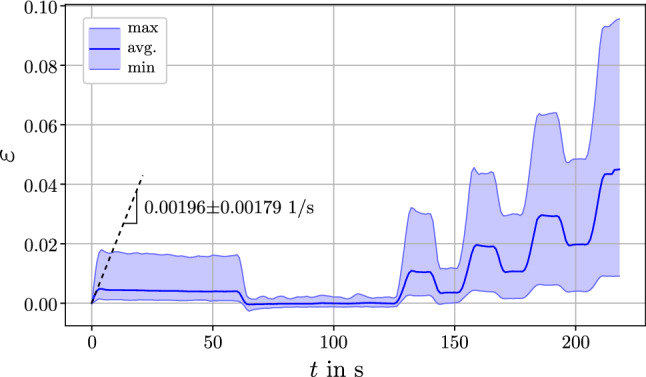
Average from all 15 strain signals obtained in the tensile tests (blue line). The shaded area represents the bandwidth of the measured strains, enclosed by the minimum (and maximum) strain value for each time point. The slope of the dotted line is the average strain rate of the first loading ramp

In total, 13 out of 28 samples had to be removed from the study due to difficulties during testing (7 cases), early fractures (2 cases), and discrepancies between strain and stress signals that became visible during data processing (4 cases). The remaining $n=15$ sets of tensile test data were further processed. Due to varying trabecular sizes and shapes, the actual strain and strain rate of the measurement length (Fig. 3d) were fluctuating among the samples, despite the applied displacement profile was identical. Figure 6 shows the strain of the measurement length averaged over all samples and its bandwidth. The average strain was obtained from averaging the strain values of all samples at each point in time. The average strain rate in the first loading ramp was found to be ${\dot{\varepsilon }}(t=2\mathrm{s})$=0.00196 ± 0.0018 1/s.

The rheological model could be optimized for the tensile test data sets with an average RMSE value of 2.91 ± 1.77MPa. For each sample around $3.5*10^6$ time series were calculated in course of the optimization procedure.

For each sample, a set of material parameters ${\underline{q}}^*$ was identified. The average and standard deviation of all sets of material parameters ${\underline{q}}^*$ are reported in Table 2 (a). The listed yield stress $\sigma _{\mathrm{Y}}$ and ultimate stress $\sigma _{\mathrm{u}}$ are already corrected for the prestress in each sample. The prestress originating from the applied preload was on average 5.52 ± 3.35 MPa.

The fit of the exponential hardening law to the experimental data resulted in an average ultimate stress of $\sigma _{\mathrm{u}}=63.99\pm 25.13$ MPa. Per definition, $\sigma _{\mathrm{u}}$ is the maximum stress level that can be reached in the Prandtl Layer by ongoing plastic deformation. This value is not to be mistaken as the failure stress of the samples. In the experiments, the samples actually failed on average at an apparent maximum stress level of $70.43\pm 26.5$MPa. This observed failure stress includes also a viscous stress contribution, whereas $\sigma _{\mathrm{u}}$ does not.

The average apparent Young’s modulus $E_{\mathrm{app}}$ was found to be higher than the modeling results, but stay in the same order of magnitude, Table 2 (b).

**Table 2:** (a) Average material parameters and their standard deviation from $n=15$ tensile trabecular samples, identified by fitting the two-layer rheological model to mechanical test data. ($\sigma _{\mathrm{Y}}$ and $\sigma _{\mathrm{u}}$ are corrected for the preload.) (b) Apparent Young’s modulus as directly extracted from stress–strain curves

(a)	Avg. ± SD	Unit
RMSE	2.91 ± 1.77	MPa
$E_{\mathrm{pr}}$	3.64 ± 2.02	GPa
$\sigma _{\mathrm{Y}}$	16.89 ± 12.67	MPa
$\sigma _{\mathrm{u}}$	63.99 ± 25.13	MPa
*p*	172.2 ± 114.0	1
$E_{\mathrm{mx}}$	1.97 ± 0.99	GPa
$\eta$	3.71 ± 3.51	GPa s
tan$(\delta )$	0.0396 ± 0.0285	1

(b)	Avg. ± SD	Unit
$E_{\mathrm{app}}$	6.32 ± 4.77	GPa

**Fig. 7:**
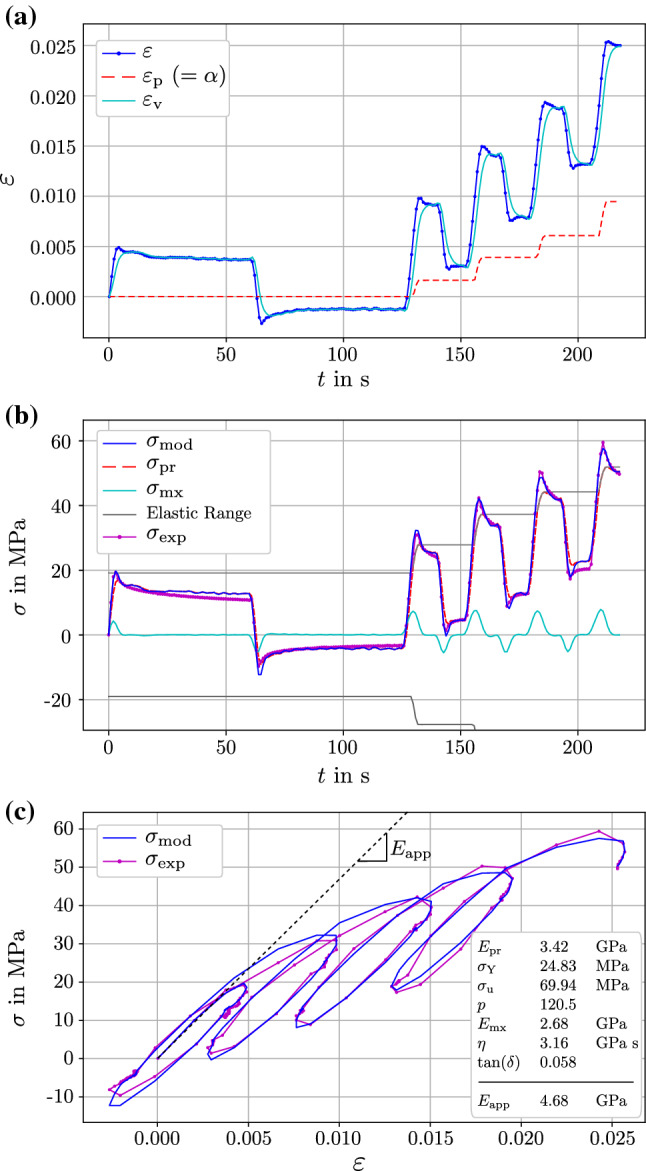
Selected tensile behavior of a single trabecular sample (id: A2439_T15) as measured experimentally (magenta) vs. simulated by the two-layer model with optimized material parameters (blue). The RMSE value for this specific sample is 1.18 MPa. **a** Strain profile $\varepsilon$ that was prescribed in the experiments and used as input signal for the two-layer model, plastic strain in the two-layer model $\varepsilon _{\mathrm{p}}$ (which is equal to the equivalent plastic strain $\alpha$ in this special case of monotonous positive plastic deformation), viscous strain $\varepsilon _{\mathrm{v}}$. **b** Stress response as measured experimentally $\sigma _{\mathrm{exp}}$ and as calculated by the two-layer model $\sigma _{\mathrm{mod}}$. The stress in the Prandtl layer $\sigma _{\mathrm{pr}}$, Maxwell layer $\sigma _{\mathrm{mx}}$ and the evolution of the elastic range is shown. **c** Stress–strain relationship comparing experiments and two-layer model and the identified material parameters for this sample

Figure 7 displays the tensile behavior of a selected sample and the according model response with the optimized set of material parameters ${\underline{q}}^*$ and the apparent stiffness $E_{\mathrm{app}}$. The RMSE of this sample is 1.18 MPa, corresponding to a good fit within the sample population.

**Fig. 8:**
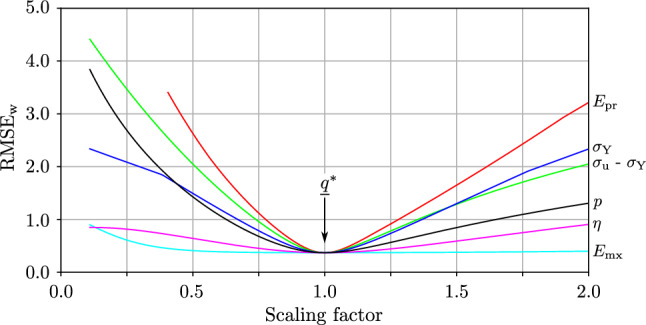
Sensitivity of the objective function $\mathrm{RMSE}_{\mathrm{w}}$ to variations in the material parameters, shown exemplary for the sample of Fig. 7. Each line shows the effect of scaling one specific material parameter, while the others were left constant at their optimum $q_i^*$ ($\widehat{=}$ scaling factor of 1.0)

For the same sample, the sensitivity of the objective function value $\mathrm{RMSE}_{\mathrm{w}}$ (Eq. 20) to variations of the material parameters was investigated. Therefore the objective function value was evaluated while varying one material parameter $q_i$ at a time, Fig. 8. The variation was performed by scaling the material parameter of interest while leaving the others at their optimum values. It is shown that for a specific relative change, the objective function is more sensitive to variations in $E_{\mathrm{pr}}$ and $\sigma _{\mathrm{Y}}$ and the least sensitive to $\eta$ and $E_{\mathrm{mx}}$.

## Discussion

In this study, a procedure for identifying elasto-visco-plastic material parameters of bone tissue from tensile test data is proposed. Therefore a two-layer rheological model is solved in the time domain with the experiment’s strain data as an input. The material parameters of the model are optimized so that the model’s stress response fits best to the experimental stress response. The resulting set of optimized material parameters are supposed to pinpoint the actual material properties of the sample. This procedure is demonstrated for tensile tests of individual bone trabeculae.

The difficulty of testing tiny trabecular specimens lead to a considerable reject rate of samples of almost 40%. This rather large number is caused mainly by sample preparation issues (7 out of 28 samples), similar as previously reported (Frank et al. 2018). In the current study, more samples had to be discarded due to discrepancies between the stress and strain signal, which might be caused by the rather long holding periods in the test protocol, combined with tiny sampling rate deviations of the camera. Two samples fractured already in the second loading cycle and were not usable for the rheological model, although the test itself worked properly. Taken together, sample preparation is the most critical part and will unfortunately always cause a rather high reject rate, as also reported in other studies (Carretta et al. 2013a; Bini et al. 2002; Hernandez et al. 2005).

*Young’s Modulus* The model could reproduce the experimental data with a good average RMSE value of 2.91 ± 1.77 MPa. The identified Young’s modulus bounds for wet single human trabeculae are $E_{\mathrm{pr}}=3.64$GPa for the long term stiffness and $E_{\mathrm{pr}}+E_{\mathrm{mx}}=5.61$GPa for the instantaneous stiffness, Table 2.

When performing mechanical tests, strain is applied neither pure quasi statically nor instantaneously but at a finite strain rate. In theory, any apparent stiffness $E_{\mathrm{app}}$ extracted from stress–strain data, obtained in such a way, should always reside within the bounds $E_{\mathrm{pr}}< E_{\mathrm{app}} < E_{\mathrm{pr}}+E_{\mathrm{mx}}$. Various methodologies for obtaining $E_{\mathrm{app}}$ exist though, such as the $R^2$- method described in this work or the maximum slope method as used in, e.g., Mirzaali et al. (2016). This methodological variation, together with potential signal noise, toe regions, etc. lead to uncertainties in determining $E_{\mathrm{app}}$. In practice $E_{\mathrm{app}}$ might therefore fall outside the bounds. This happened in this work, where - on average - $E_{\mathrm{app}}$ is larger than $E_{\mathrm{pr}}+E_{\mathrm{mx}}$, Table 2.

In the context of other studies on micro-tensile tests of single trabeculae, this study’s $E_{\mathrm{pr}}$ and $E_{\mathrm{pr}}+E_{\mathrm{mx}}$ appear to be within the wide range of reported values. Frank et al. (2017, 2018) tested wet bovine trabeculae in two studies in tension and reported an average Young’s modulus of 8.2 GPa and 6.5 GPa, respectively. Other works were performed dry, resulting in significantly higher stiffness values of 10.4 GPa for human trabeculae (Rho et al. 1993), 11.84 GPa for a young and 15.56 GPa for an old bovine source (Carretta et al. 2013a), and around 16.5 GPa for human femoral trabeculae (Carretta et al. 2013b). The current results are in good agreement with the work of Choi et al. (1990), who tested moist cortical- and trabecular specimens and found their Young’s modulus to be around 4.59 GPa and 5.44 GPa, respectively. Likewise, Szabó et al. (2011b) reported 5.2 ± 3.1 GPa from three point bending tests on fully hydrated bovine trabeculae. Bini et al. (2002) obtained values of 1.41–1.89 GPa on human femoral struts which is surprisingly low for dry conditions. Ryan and Williams (1989) tested bovine trabeculae and also found them to be compliant with 0.4–1.8 GPa.

As shown in Fig. 8 for a specific sample, the instantaneous bone stiffness is tentatively subjected to a higher uncertainty as the optimization process is considered less sensitive to $E_{\mathrm{mx}}$. Interestingly, even high variations of $E_{\mathrm{mx}}$ would impose almost no change in the model’s goodness of fit. This could be attributed to the relatively low stress contribution of the Maxwell layer to the total model stress, Fig. 7b.

*Yield stress* The yield stress $\sigma _{\mathrm{Y}}$ of a material is defined as the stress level at which the first plastic deformation occurs when a sample is loaded quasi-statically and monotonically. In theory this is the last point of the initial linear region in stress–strain data. There are multiple approaches for determining the apparent yield stress of bone from mechanical testing data. Sometimes the linear region is being determined by the $R^2$ method, see also Sec. 2.4, with its last point taken as the yield stress. Other methods use the 0.2% strain limit (Keaveny et al. 1994a), or a line intersection method (Reilly and Burstein 1975), that likely would produce different results. The mentioned methods are also hard to apply, if the loading protocol is non-monotonic.

In addition to the methodological ambiguities, mechanical tests are in general not really quasi-static. The resulting force-displacement data includes necessarily a deceptive viscous force contribution depending on the applied strain rate. The yield stress $\sigma _{\mathrm{Y}}$ obtained from the two-layer model is excluding viscous effects and represents a pure quasi-static quantity. From the theoretical point of view, an apparent yield point, extracted directly from force–displacement data, is therefore in general higher than the pure $\sigma _{\mathrm{Y}}$ of the two-layer model.

In this study, the apparent yield stress was not extracted from the stress strain curve, as the first loading ramp was too low for some samples to allow for a robust determination. However, the found $\sigma _{\mathrm{Y}}$ in this work of 16.89 ± 12.67MPa is substantially lower than values from other studies, where yield stresses of 60–80 MPa were found in wet micro-tensile tests on bovine trabeculae, extracted by curve fitting (Frank et al. 2017, 2018). Carretta et al. (2013a) reported even higher yield stresses between 78 MPa and 115 MPa as measured on dry bovine trabeculae and in Carretta et al. (2013b). 115–130 MPa for dry human femoral trabeculae were obtained. Tensile tests on wet compact bone specimens provide a similar yield limit of 122.3 MPa (Currey 2004). To conclude, the obtained yield stresses of the present study appear to be low in the context of other literature. Comparability is currently limited though, as no study exists, that reports the yield stress of single wet (submerged) human trabeculae measured really quasi-statically.

*Viscosity* The two-layer model allows for a direct evaluation of the loss tangent $\tan (\delta )$ based on $E_{\mathrm{pr}}, E_{\mathrm{mx}}, \eta$ and a chosen frequency. The loss tangent is proportional to the ratio of dissipated to stored energy for harmonic cyclic loading. Samples were obtained from a human femur, so the cyclic loading of walking was considered physiologically relevant. For the sake of simplicity, a walking frequency of 1Hz was assumed and chosen for evaluating $\tan (\delta )$. Hereby an average $\tan (\delta )=0.04\pm 0.029$ was obtained.

To the authors knowledge, no study investigated this property on wet single trabeculae. One lengthscale above, at the level of compact bone, Garner et al. (2000) did torsional and bending tests on human specimens in wet conditions and obtained values between 0.01 and 0.03 for $\tan (\delta )$ at 1 Hz. Similar values of 0.013 for torsional tests on wet bovine bone at 1 Hz are reported by Lakes et al. (1979). Yamashita et al. (2001) obtained an result of $\tan (\delta )=0.042\pm 0.006$ from DMA on wet, millimeter sized, human femoral cortical bone samples at 1 Hz. In summary, the loss tangent obtained in this study on individual trabeculae is in good accordance with experiments performed on compact bone.

**Fig. 9:**
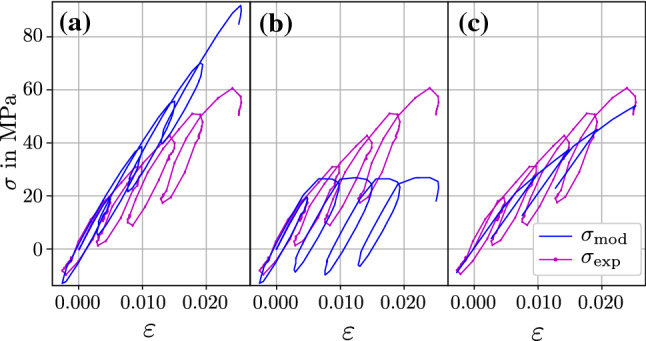
Three cases, showing the consequences of switching off constitutive effects of the two-layer model, each based on the model response shown in Fig. 7c. **a** No plasticity ($\sigma _{\mathrm{Y}}=\infty$), **b** no hardening ($\sigma _{\mathrm{u}}=\sigma _{\mathrm{Y}}$) and **c** omitted Maxwell layer ($E_{\mathrm{mx}}=0$, $\eta =0$)

**Fig. 10:**
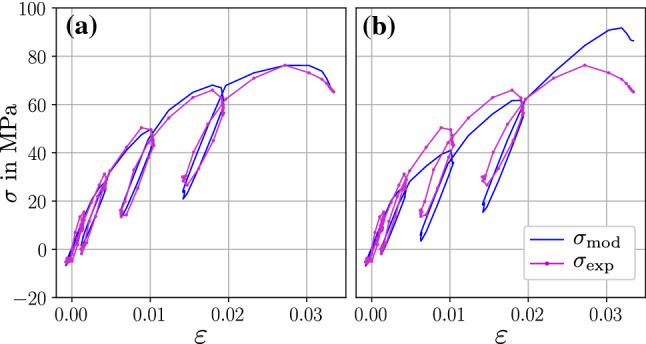
Consequences of switching from exponential hardening (**a**) to linear hardening (**b**), demonstrated for the model response for sample id: A2439_T21. The linear hardening coefficient was also optimized to fit best. The RMSE value increases from 2.17 MPa (**a**) to 5.67 MPa (**b**)

*Model performance* The challenge of parameter identification increases with the number of parameters to optimize. Some elasto-visco-plastic rheological models for bone exist that are more complex than the proposed two-layer model, e.g., Peric and Dettmer (2003), Schwiedrzik et al. (2014); Schwiedrzik (2014). Due to a larger parameter count, they allow for more flexibility. However, having a low number of parameters is crucial to minimize computational expense and finding as unique solutions as possible. This leads to the question if the proposed two-layer topology of Fig. 1 is the simplest form to reflect the trabecular tensile characteristics or if it could be simplified further while keeping the goodness of fit. This was addressed qualitatively by switching of constitutive elements of the two-layer model in alternation and investigating the changes in its response. Figure 9 shows that exemplary for the sample of Fig. 7 (id: A2439_T15). It can be seen that locking plastic deformation leads to an overestimation of stress. On the other hand, using perfect plasticity instead of exponential hardening misses the gradual stress increase for each loading cycle. When omitting the viscous layer, the hysteresis loop that occurs in each loading cycle cannot be modeled. As displayed for another selected sample (id: A2439_T21) in Fig. 10, switching from exponential hardening to linear hardening downgrades the model fit. The latter effect varies among the samples and is stronger for higher post-yield strains.

Despite the few rheological elements in the model, a good fit could be reached for the presented difficult microtensile tests. It is therefore concluded that the proposed two-layer topology is sufficiently complex—but not more—to reproduce the elasto-visco-plastic mechanical response of bone trabeculae.

The used cyclic loading protocol attempted to make the constitutive effects of elasticity, plasticity, and viscosity visible and to ease the optimization procedure. Preliminary optimization trials with an unweighted objective RMSE function, where the weight factors are $w_i=1.0$ at all data points, lead often to a bad fit. Although the obtained RMSE values were similarly low as in Table 2, the model response was often not reproducing the characteristic corner points of the loading cycles but showed an indistinct and diffuse profile. The implementation of the weighted $\hbox {RMSE}_{\mathrm{w}}$ where $w_i=1.0$ at the corners and $w_i=0.0$ otherwise, forced the model response to cover the corner points of the loading cycles and improved the optimization results significantly. On the downside, this indicates that the material parameter identification results are depending on a proper selection of the objective function and are not robust in this regard.

The two-layer model disregards the constitutive effect of damage in the sense of stiffness reduction induced by plastic deformation (Burr et al. 1998; Garcia et al. 2010). Compact bone on the microscale appears to show hardly any damage (Schwiedrzik et al. 20140, whereas on the tissue level of trabecular bone, damage evolves with the amount of fractured trabeculae (Keaveny et al. 1994b; Zysset and Curnier 1996). At the lengthscale of single trabeculae, damage is associated with microcracks whose size and density were shown to increase with plastic strain (Jungmann et al. 2011; Frank et al. 2018). The damaged zones are thereby observed as whitening. Ridha and Thurner (2013) quantified the damage factor and -exponent for single trabeculae in 3 point bending conditions. Applying these damage parameters on the average plastic strains observed in this study, the reduction in stiffness would be below 1%. Therefore it seems justified to neglect the effect of damage in the current work. For the same reason, the two-layer model should be suitable to be applied on other sufficiently small bone specimens like micro pillars or millimeter sized compact bone samples.

**Fig. 11:**
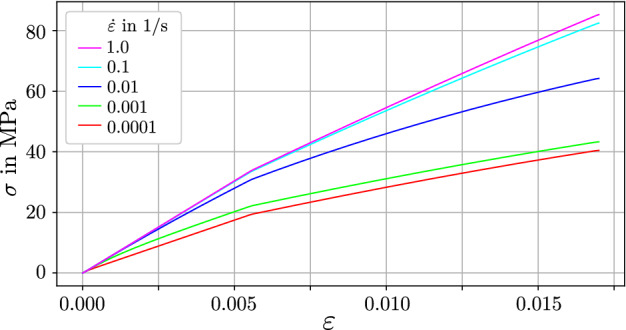
The stress response of the two-layer model at different strain rates between ${\dot{\varepsilon }}=$ 0.0001–1.0 1/s. The model is set to the material parameters of Fig. 7. The graph is to be related to similar figures from, e.g., McElhaney (1966) or Johnson et al. (2010)

*Strain-rate-dependent apparent properties* Multiple studies showed that the apparent Young’s modulus of bone as well as the levels of apparent yield stress and apparent ultimate stress are positively correlated with strain rate (McElhaney 1966; Currey 1975; Hansen et al. 2008; Johnson et al. 2010). The two-layer topology is in general able to capture that behavior: In Fig. 11 the two-layer model configured with the material parameters from the demonstration sample of Fig. 7 is subjected to a ramp loading at different strain rates. The apparent Young’s modulus, indicated by the initial slope, as well as apparent yield stress, indicated by the stress level of the kink in the curve are increasing with strain rate. Interestingly, the highest changes are observed within the range of physiological strain rates between 0.001 and 0.1 1/s (Hansen et al. 2008).

This positive correlation between strain rate and apparent stiffness and -yield stress of bone tissue could not yet be confirmed at the level of individual bone trabeculae. To the authors knowledge, only Szabó et al. (2011a) attempted to shed some light on this question and tested hydrated trabeculae in three point bending mode at different speeds spanning almost three orders of magnitude. Contrary to expectations, the obtained Young’s moduli were almost not affected by the variations in strain rate.

*Limitations* First, the formulation of the two-layer model is valid for geometrically linear problems only. An approach for elasto-visco-plastic rheological models at large strains is covered in Kiessling et al. (2016). In the outlined form, the two-layer model is utilizing the measures of engineering stress and strain and is limited to geometrically linear deformations. However, strains up to 9% were applied on some of the trabeculae, Fig. 6. For this strain level and an estimated Poisson’s ratio of 0.25 for bone micro-structural units, taken from Reisinger et al. (2010), the engineering stress is underestimating the true stress by approx. 4%. The conjugated logarithmic strain would give 8.6% vs. 9% of engineering strain at that stage. These errors increase with even larger strains.

Second, the proposed optimization approach utilized a multi-start method in order to increase the chance of finding the global solution of the optimization problem. In addition, the loading profile attempted to accentuate viscosity and plasticity to ease the numerical evolution of the optimization process and to increase the *uniqueness* of the solution. In the course of this, $4^6=4096$ solutions for each sample were obtained from which the one with the lowest $\hbox {RMSE}_{\mathrm{w}}$-value was picked. The question of uniqueness was not further addressed. However, solutions with a slightly worse $\hbox {RMSE}_{\mathrm{w}}$-value and a significantly different set of material parameters ${\underline{q}}$ might exist, that should be further investigated.

## Conclusion

A two-layer elasto-visco-plastic rheological model is presented, capable of reproducing the stress response of single trabeculae subjected to uniaxial cyclic loading. The model is applied on stress–strain data in an inverse approach, to identify stiffness, yield, and viscous material parameters. These are—where comparable—in meaningful accordance with conventionally obtained parameters and results from similar studies.

The presented procedure is supposed to be applicable on other materials as well if they show a similar elasto-visco-plastic behavior.

It was shown, that the proposed two-layer model alongside with the optimization approach can be of great advantage when multiple constitutive effects shall be quantified based on a single mechanical measurement. This is especially useful in the field of bone mechanics, where each specimen is unique and is usually tested only once.

## Appendix: Solution of the Prandtl layer

The problem to be solved in the following, consists of determining the stress in the Prandtl layer $\sigma _{\mathrm{pr}}$ for a given strain signal $\varepsilon$. We sum up the problem in the form of a system of ODEs

23 $$\begin{aligned} \dot{{\underline{y}}}(t) = {\underline{g}}(t,{\underline{y}}(t)) \quad \mathrm{with} \quad {\underline{y}}= \begin{bmatrix} \sigma _{\mathrm{pr}} \\ \alpha \end{bmatrix} \end{aligned}$$

with ${\underline{g}}$ being a vector function, comprising of Eqs. 3, 4, 5, 6, 11.

The ODE in Eq. 23 is stiff in nature due to the discontinuous Eq. 11. Nevertheless, it can be solved by a standard ODE solver, using e.g. the Backward Differentiation Formula (BDF) (Byrne and Hindmarsh 1975), and by allowing $f(\sigma _{\mathrm{pr}},\alpha )$ to take ’small’ values greater than 0. However, this is highly inefficient.

More favorably, the *return mapping algorithm* for the incremental solution of $\sigma _{\mathrm{pr}}$ is employed (Simo 1998). Therefore, time and strain are discretized

24 $$\begin{aligned} t_{i+1}=t_i+{\varDelta } t \quad \mathrm{and} \quad \varepsilon _{i+1}=\varepsilon _i+{\varDelta } \varepsilon \end{aligned}$$

with the time increment ${\varDelta } t$ and strain increment ${\varDelta } \varepsilon$ advancing time and strain from a state *i* to the state $i+1$ ($i \in {\mathbb{N}}$). Solving the problem of Eq. 23 then reduces to finding the model state ${\underline{y}}_{i+1}$ coming from a known solution ${\underline{y}}_{i}$. The new state ${\underline{y}}_{i+1}$ can be approximated by different integration schemes, from which the *backward (implicit) Euler* algorithm is used for the reason of its unconditional stability (Hairer et al. 1987). In that framework, the discrete version of Eq. 23 reads

25 $$\begin{aligned} {\underline{y}}_{i+1} = {\underline{y}}_{i} + {\varDelta } t \ {\underline{g}}(t_{i+1},{\underline{y}}_{i+1}) \end{aligned}$$

The algorithmic counterparts of Eqs. 3, 4, 5, 6 are

26 $$\begin{aligned} \begin{aligned} \sigma _{\mathrm{pr}, i+1}&= E_{\mathrm{pr}} (\varepsilon _{i+1} - \varepsilon _{\mathrm{p}, i+1}), \\ \varepsilon _{\mathrm{p}, i+1}&= \varepsilon _{\mathrm{p}, i} + {\varDelta } \gamma \ \mathrm{sign}(\sigma _{\mathrm{pr}, i+1}), \\ \alpha _{i+1}&=\alpha _i+{\varDelta } \gamma , \\ f_{i+1}&= |\sigma _{\mathrm{pr}, i+1}| - [\sigma _{\mathrm{Y}}+(\sigma _{\mathrm{u}}-\sigma _{\mathrm{Y}})(1-\exp (-\alpha _{i+1} p))] \end{aligned} \end{aligned}$$

alongside with the discrete version of the Kuhn–Tucker conditions

27 $$\begin{aligned} \begin{aligned} f_{i+1}&\le 0, \\ {\varDelta } \gamma&\ge 0, \\ {\varDelta } \gamma \ f_{i+1}&= 0 \end{aligned} \end{aligned}$$

where ${\varDelta } \gamma = \gamma _{i+1} {\varDelta } t$ is a Lagrange multiplier.

The return mapping strategy involves conducting an elastic *trial* step while freezing plastic flow, defined by

28 $$\begin{aligned} \begin{aligned} \sigma _{\mathrm{pr}, i+1}^{\mathrm{trial}}&= E_{\mathrm{pr}} (\varepsilon _{i+1} - \varepsilon _{\mathrm{p}, i}), \\ \varepsilon _{\mathrm{p}, i+1}^{\mathrm{trial}}&= \varepsilon _{\mathrm{p}, i}, \\ \alpha _{i+1}^{\mathrm{trial}}&= \alpha _{i}, \\ f_{i+1}^{\mathrm{trial}}&= |\sigma _{\mathrm{pr}, i+1}^{\mathrm{trial}}| - [\sigma _{\mathrm{Y}}+(\sigma _{\mathrm{u}}-\sigma _{\mathrm{Y}})(1-\exp (-\alpha _{i+1}^{\mathrm{trial}} p))] \end{aligned} \end{aligned}$$

In case, the trial step is admissible in the sense that $f_{i+1}^{\mathrm{trial}} \le 0$, the trial step *is* in fact the solution to the problem (Eqs. 26, 27). This step is called a purely *elastic step* and ${\varDelta } \gamma =0$, giving

29 $$\begin{aligned} \left. \begin{array}{c} \sigma _{\mathrm{pr}, i+1} =\sigma _{\mathrm{pr}, i+1}^{\mathrm{trial}}, \\ \\ \varepsilon _{\mathrm{p}, i+1} = \varepsilon _{\mathrm{p}, i+1}^{\mathrm{trial}}, \\\\ \alpha _{i+1} = \alpha _{i+1}^{\mathrm{trial}}, \\ \\ f_{i+1} = f_{i+1}^{\mathrm{trial}} \end{array}\right\} \mathrm{if} \ f_{i+1}^{\mathrm{trial}} \le 0 \end{aligned}$$

In the other case, where the trial step resides outside the yield surface (indicated by $f_{i+1}^{\mathrm{trial}} > 0$) the step under consideration is not admissible in the sense of Eq. 27. The solution to this *plastic step* needs to be obtained by ’correcting’ the trial step by

30 $$\begin{aligned} \left. \begin{array}{l} \sigma _{\mathrm{pr}, i+1} =\sigma _{\mathrm{pr}, i+1}^{\mathrm{trial}} - {\varDelta } \gamma \ E_{\mathrm{pr}} \ \mathrm{sign}(\sigma _{\mathrm{pr}, i+1}^{\mathrm{trial}}) , \\ \varepsilon _{\mathrm{p}, i+1} = \varepsilon _{\mathrm{p}, i+1}^{\mathrm{trial}} + {\varDelta } \gamma \ \mathrm{sign}(\sigma _{\mathrm{pr}, i+1}^{\mathrm{trial}}), \\ \alpha _{i+1} = \alpha _{i+1}^{\mathrm{trial}} + {\varDelta } \gamma , \\ f_{i+1} \equiv 0 \end{array}\right\} \mathrm{if} \ f_{i+1}^{\mathrm{trial}} > 0 \end{aligned}$$

Finally, breaking down the requirement $f_{i+1} \equiv 0$ gives the implicit relationship

31 $$\begin{aligned} \begin{aligned}&f_{i+1} = |\sigma _{\mathrm{pr}, i+1}^{\mathrm{trial}}| - {\varDelta } \gamma \ E_{\mathrm{pr}} \\&- [\sigma _{\mathrm{Y}}+(\sigma _{\mathrm{u}}-\sigma _{\mathrm{Y}})(1-\exp (-p (\alpha _{i}+{\varDelta } \gamma )))] \equiv 0 \end{aligned} \end{aligned}$$

or, in short, with Eq. 4

32 $$\begin{aligned} f(\sigma _{\mathrm{pr}, i+1}^{\mathrm{trial}},\alpha _{i}+{\varDelta } \gamma )- {\varDelta } \gamma \ E_{\mathrm{pr}}=0 \end{aligned}$$

from which the only remaining unknown ${\varDelta } \gamma > 0$ can be obtained numerically via, e.g., the Newton–Raphson algorithm.
