# Correction to: A two‑layer elasto‑visco‑plastic rheological model for the material parameter identification of bone tissue

**DOI:** 10.1007/s10237-020-01356-x

**Published:** 2020-06-24

**Authors:** Andreas G. Reisinger, Martin Frank, Philipp J. Thurner, Dieter H. Pahr

**Affiliations:** 1 Division Biomechanics, Department of Anatomy and Biomechanics, Karl Landsteiner University of Health Sciences, Krems an der Donau, Austria; 2 Institute of Lightweight Design and Structural Biomechanics, Vienna University of Technology, Vienna, Austria

## Correction to: Biomechanics and Modeling in Mechanobiology 10.1007/s10237-020-01329-0

In the original publication of the article, Eq. (29) was incorrectly published as given below:

$$ \left. {\begin{array}{l} {\sigma_{{{\text{pr}},i + 1}} = \sigma_{{{\text{pr}},i + 1}}^{{{\text{trial}}}}} -\varDelta \gamma E_{\text{pr}}{\text{sign}} (\sigma_{{\text{pr},}i + 1}^{\text{trial}}) , \hfill \\ {\varepsilon_{{{\text{p}},i + 1}} = \varepsilon_{{{\text{p}},i + 1}}^{{{\text{trial}}}}} +\varDelta \gamma {\text{sign}} (\sigma_{{\text{pr},}i + 1}^{\text{trial}}), \hfill \\ {\alpha_{i + 1} = \alpha_{i + 1}^{{{\text{trial}}}}} + \varDelta \gamma, \hfill \\ {f_{i + 1}\equiv 0}  \hfill \\ \end{array} } \right\}\;{\text{if}}\;f_{i + 1}^{{{\text{trial}}}} > 0 $$

However, Eq. (29) should be as given below:

$$ \left. {\begin{array}{l} {\sigma_{{{\text{pr}},i + 1}} = \sigma_{{{\text{pr}},i + 1}}^{{{\text{trial}}}} ,} \hfill \\ {\varepsilon_{{{\text{p}},i + 1}} = \varepsilon_{{{\text{p}},i + 1}}^{{{\text{trial}}}} ,} \hfill \\ {\alpha_{i + 1} = \alpha_{i + 1}^{{{\text{trial}}}} ,} \hfill \\ {f_{i + 1} = f_{i + 1}^{{{\text{trial}}}} } \hfill \\ \end{array} } \right\}\;{\text{if}}\;f_{i + 1}^{{{\text{trial}}}} \le 0 $$

The original article has been corrected.

